# Surgery for localized pulmonary mycotic infections in patients with hematopoietic disorder

**DOI:** 10.1186/s13019-015-0297-7

**Published:** 2015-06-30

**Authors:** Youngkyu Moon, Jae Kil Park, Sook Whan Sung

**Affiliations:** Department of Thoracic & Cardiovascular Surgery, Seoul St. Mary’s Hospital, College of Medicine, The Catholic University of Korea, 222 Banpo-daero, Seocho-gu, Seoul, 137-701 Republic of Korea

**Keywords:** Lung disease, Fungal, Pulmonary surgical procedures, Complications

## Abstract

**Background:**

Surgical resection is considered to be the most effective treatment for localized pulmonary mycotic infections. However it is also a particularly challenging procedure because it is associated with considerable mortality and morbidity. Furthermore, hematopoietic disorders usually cause immunosuppression, anemia, and coagulopathy, which are definite risk factors for surgery. The purpose of this study is to evaluate the surgical outcomes of pulmonary mycotic infections in hematopoietic disorder patients.

**Methods:**

Between 2011 and 2013, 23 patients underwent surgical treatment for pulmonary mycotic infections at a single institution. The patients were divided into two groups; Group A (hematopoietic disorder patients, *n* = 9) and Group B (*n* = 14). We retrospectively reviewed medical and radiologic data.

**Results:**

The complex type was more frequent in group A (66.6 %) than in group B (35.7 %). Postoperatively, there was no mortality. However, morbidity was 22.2 % (2 incomplete expansion) in group A, and 35.6 % (1 prolonged air leak, 3 bleeding, 1 Bronchopleural fistula) in group B. The difference in morbidity between the groups did not show any statistical significance (*p* = 0.657) as well as duration of chest tube drainage, and postoperative hospital stay. The hematopoietic disorder patients did not impose a risk factor for morbidity and mortality.

**Conclusions:**

Although hematopoietic disorder patients have many surgical risk factors, the surgical treatment of pulmonary mycotic infections produces very acceptable outcomes in selected cases.

## Background

Hematopoietic disorder is a common designation for hematopoiesis-related diseases such as aplastic anemia, myelodysplastic syndrome, and leukemia [1]. Because they often suffer from immunosuppression, anemia and/or coagulopathy, patients with this disorder are considered to be high-risk factor for operation [2]. The immunosuppression of these patients could be caused by the underlying disease itself, but in many cases it has intensified after chemotherapy treatment or hematopoietic stem cell transplantation (HSCT). Fungal infection is more likely to occur if neutropenia continues after chemotherapy or HSCT [3]. Fungal disease occurring in this type of patient is often invasive. Even though it can be initially diagnosed as localized disease, it can also be detected as extended disease from the beginning, and when not treated, mortality has been reported as high as 100 % due to dissemination [3]. Despite the use of antifungal agents in the treatment of all patients with invasive fungal infection, a high rate of mortality has nevertheless been reported, ranging from 20 to 40 % [4, 5], and it is recognized that it is difficult in practice to achieve complete clearance of fungal infection by antifungal agent therapy alone [4, 6]. Therefore, the most effective treatment is considered to be surgical clearance of fungus before HSCT, or if persistent immunosuppression is expected. However, a decision as to whether to perform surgery can be difficult due to the high surgical risk.

Various fungi can cause pulmonary mycotic infections, of which Aspergillus infection is the most frequent [7]. Of the many different forms of Aspergillus infection, allergic bronchopulomonary aspergillosis (ABPA), aspergilloma, and invasive pulmonary aspergillosis (IPA) are the most commonly found [7]. Among these, aspergilloma mainly manifests as a localized disease occurring in underlying lung disease. IPA outbreaks occur mostly in immunocompromised patients in the form of a localized or extended disease. In case of aspergilloma, many reports have confirmed the effectiveness of operative treatment, though the operative risk is somewhat high since in many cases the underlying lung disease is severe [8–10]. However, in the case of IPA, surgical treatment may be difficult to perform in many cases due to the high rate of parenchymal invasion. Despite reports of surgical treatment in cases where it has occurred as a localized disease, the paucity of such cases has given rise to considerable uncertainty as to the effectiveness of surgical treatment [11–14].

The purpose of this study was to evaluate the possibility of surgical treatment for hematopoietic disorder patients by analyzing the differences in operative risk and the outcome of surgical treatments between the general localized pulmonary mycotic infection and the localized pulmonary mycotic infection occurring in hematopoietic disorder patients with high operative risk.

## Methods

A retrospective chart review was conducted on 23 patients who underwent surgical treatment for pulmonary mycotic infections at Seoul St Mary’s Hospital between 2011 and 2013. In order to evaluate the surgical outcomes of hematopoietic disorder patients, the patients were divided into two groups; Group A (hematopoietic disorder patients, *n* = 9) and Group B (other patients, *n* = 14), and the characteristics and surgical outcomes of the two groups were compared. The underlying diseases of Group A were: acute myeloid leukemia (AML) (*n* = 3), acute lymphoblastic leukemia (ALL) (*n* = 3), myelodysplastic syndrome (MDS) (*n* = 1), and aplastic anemia (*n* = 2). Those of Group B were: bronchiectasis (*n* = 4), old tuberculosis (*n* = 4), end stage renal disease (*n* = 1), liver cirrhosis (*n* = 1) and non-specific underlying disease (*n* = 4).

Open thoracotomy or video-assisted thoracoscopic surgery (VATS) was performed, as was pulmonary resection, wedge resection, segmentectomy, or lobectomy, depending on the extent of the disease, as a curative procedure.

In the pre-surgery diagnosis, chest computed tomography (CT) scan played the most important role, and in cases where the ‘halo sign’ or ‘air-crescent sign’ was detected, early surgical resection treatment was performed when there was a strong suspicion of aspergilloma. However, in cases when atypical pneumonic consolidation was detected, surgical treatment was only performed on patients if complete surgical resection became feasible after antifungal agent therapy and in correlation with the clinical information.

Through histologic examination after surgery, mycotic infection was confirmed in all cases, there being 21 cases of aspergillosis and 2 cases of mucormycosis. In cases of Aspergillus infection, aspergilloma was diagnosed separately from IPA by confirming bronchopulmonary invasion, vessel invasion, or pulmonary infarction in the pathologic findings.

**Fig. 1:**
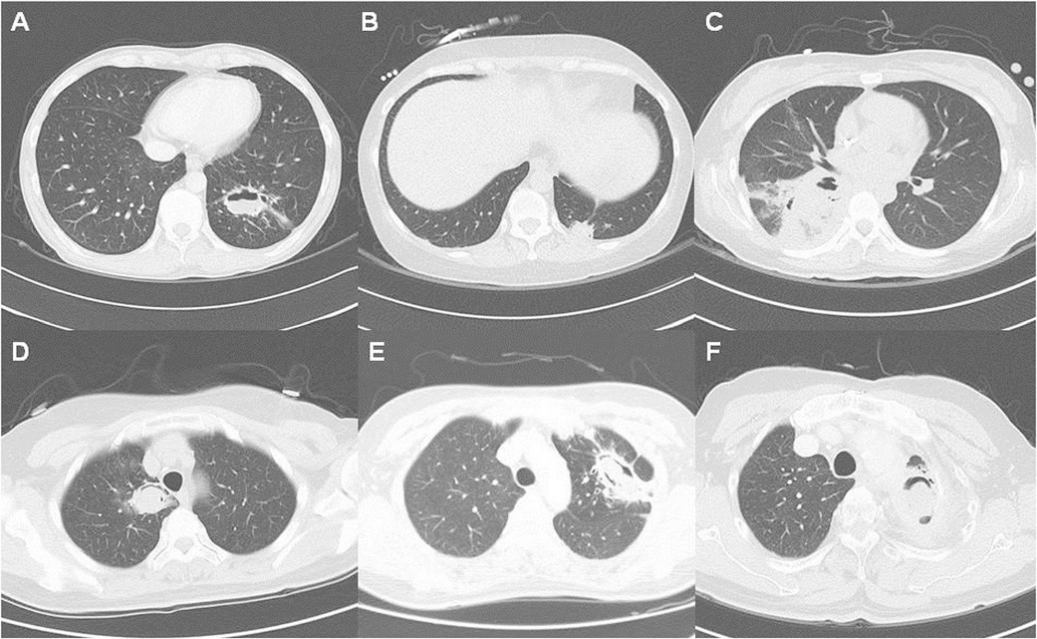
**a** Simple aspergilloma (*Group A*) **b** mucormycosis (*Group A*) **c** invasive pulmonary aspergillosis (*Group A*) **d** simple aspergilloma (*Group B*) **e** complex aspergilloma (*Group B*) **f** complex aspergilloma with empyema (*Group B*)

Aspergilloma occurred often in the form of a fungus ball and was categorized into simple and complex types by the shape of its lesion. Aspergilloma was classified as the simple type in cases where the surrounding lung was comparatively normal and the capsule was thin. It was classified as the complex type if the lung surrounding the lesion was diseased or the capsule was thick [15]. However, cases of mucormycosis and IPA were not classified into the types previously referred to in existing studies. In this study, the types of Group B patients (*n* = 14, all with aspergilloma) were classified in the same way by applying the pertinent type definition to the shape of the lesion as shown on the chest CT image. Out of the 9 patients in Group A, there were 1, 2, and 6 patients who were pathologically diagnosed as aspergilloma, mucormycosis, and IPA respectively. A patient with aspergilloma was classified as a simple type as per the CT image, and 2 patients with mucormycosis were also classified as simple types since both of them showed a single nodule with the lesion’s surrounding lung parenchyma showing a normal shape on CT image. Six patients with IPA were classified as complex types since their parenchymal lesion was accompanied by infiltration and there were many similarities to the complex type as per the CT image. Thereafter the types were matched between the two groups (Fig. 1).

Follow-up data was obtained by review of the outpatient charts. Chi-square or t test was used for the comparison of preoperative, intraoperative, and postoperative factors of the two groups and a value of *P* < 0.05 was considered statistically significant. Operative mortality was defined as in-hospital mortality or those cases in which the patient died within 30 days following surgery. The relapse rate after operation was counted and the all-cause mortality rate was evaluated. Logistic regression analysis was used to evaluate the risk factor of postoperative complications. The study was approved by the institutional Review Board of Seoul St. Mary’s Hospital (The Catholic University of Korea).

## Results

There were 9 patients in Group A and 14 patients in Group B, making a total of 23 patients. The mean age of Group A was 34.7 years (range 14–63) and of Group B, 49.1 years (range 20–69). The mean age of Group A was lower than that of Group B (*p* = 0.043). Females outnumbered males in Group A with 6 female patients (66.6 %) and 3 male patients (33.4 %); while in Group B the number of males and females was the same with 7 patients (50 %) of each sex. Regarding preoperative symptoms, in Group A fever was the most common with 6 instances (66.6 %), and 1 subsequent instance (11.1 %) of hemoptysis, cough, and subclinical. In Group B hemoptysis was the most frequent sympton with 7 instances (50 %) and subsequently there were 5 instances of subclinical (35.7 %) and 2 instances (14.3 %) of cough. Even though white blood cell (WBC) counts and absolute neutrophil counts (ANCs) of both groups in the preoperative complete blood cell count (CBC) did not show significant differences, both the hemoglobin and platelet counts of Group A were significantly less (9.8(±1.4)g/dl vs 12.2(±2.0)g/dl, *p* = 0.006), (112.2(±72.2) × 10^9^/L vs 201.5(±78.8) × 10^9^/L, *p* = 0.012). In pulmonary function testing, there were no differences in the two groups in forced expiratory volume in 1 s (FEV1) and forced vital capacity (FVC). In the type classification by image findings, there were 3 patients (33.3 %) of simple type and 6 patients (66.6 %) of complex type in Group A, and there were 9 patients (64.3 %) of simple type and 6 patients (35.7 %) of complex type in Group B. Even though the complex type ratio was higher in Group A, it was not statistically significant (*p* = 0.147) (Table 1).

**Table 1:** Patients characteristics

	Group A (*n* = 9)	Group B (*n* = 14)	P value
Age	34.7 (±16.6)	49.1 (±15.0)	0.043
Gender			
Male	3 (33.3 %)	7 (50 %)	
Female	6 (66.6 %)	7 (50 %)	
Symptom			
Hemoptysis	1 (11.1 %)	7 (50 %)	
Fever	6 (66.6 %)	0 (0 %)	
Cough	1 (11.1 %)	2 (14.3 %)	
None	1 (11.1 %)	5 (35.7 %)	
WBC count (×10^6^/L)	5972.2 (±4973.4)	7064.3 (±2720.7)	0.502
ANC count (×10^6^/L)	4335.0 (±4388.9)	4450.7 (±2402.9)	0.935
Hemoglobin (g/dl)	9.8 (±1.4)	12.2 (±2.0)	0.006
Platelet count (×10^9^/L)	112.2 (±72.2)	201.5 (±78.8)	0.012
PEV1 (%)	93.2 (±7.9)	96.3 (±15.1)	0.676
Type			
Simple	3 (33.3 %)	9 (64.3 %)	0.147
Complex or IPA	6 (66.6 %)	5 (35.7 %)	

*WBC* white blood cell, *ANC* absolute neutrophil count, *FEV1* forced expiratory volume in 1 s

**Fig. 2:**
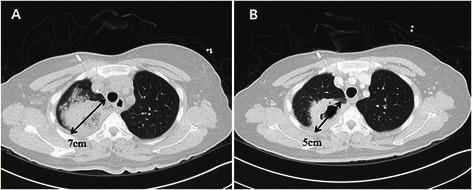
Size reduction after antifungal medical treatment. **a** Invasive pulmonary aspergillosis of which diameter was 7 cm in the right upper lobe. **b** Size reduction to 5 cm after administration of intravenous amphotericin B for 24 days

Before operation, all patients in Group A were treated with antifungal agents, but Group B patients were not. Antifungal agents used in Group A were amphotericin B for 4 patients, IV voriconazole for 2 patients, and oral antifungal agent (fluconazole, itraconazole, posaconazole) for 3 patients, with the average period of treatment 44.3 days (16–147 days). Six patients (66.7 %) showed a partial response such as reduction of infiltration or size in the surrounding lung (Fig. 2).

The surgical procedure was different depending on the range of the disease and the operation was performed for complete resection of the localized lesion. In the case of Group A, 2 procedures were performed on 3 patients out of the 9, making a total of 12 procedures in all. In the case of Group B, 2 procedures were performed on 4 patients out of 14, making a total of 18 procedures in all. In both groups lung wedge resection was the most frequently performed procedure with segmentectomy and lobectomy next in frequency. In cases of simple type, pulmonary lesions were easy to resect without 1 case of enucleation performed on a right upper lobe (RUL) nodule close to a RUL bronchus in Group B. However, in cases of complex type including invasive aspergillosis, pulmonary resections were difficult due to severe adhesion, fragility of lung parenchyme, and unresectable inflammatory lymph nodes. In some cases of complex type, other procedures were needed: 1 case of myocardial abscess drainage performed along with lung wedge resection in Group A, 1 case of cavernostomy performed on a fungus ball with chronic pleural empyema in Group B. The spectrum of surgical procedures did not differ significantly between the two groups (*p* = 0.500) (Table 2).

**Table 2:** Surgical procedures

	Group A (*n* = 12)	Group B (*n* = 18)	*P* value
Procedure			0.500
Lobectomy	4 (33.3 %)	2 (11.1 %)	
Segmentectomy	1 (8.3 %)	3 (16.7 %)	
Wedge resection	6 (50.0 %)	11 (61.1 %)	
Other procedures	1 (8.3 %)	2 (11.1 %)	
VATS	5 (55.6 %)	11 (78.6 %)	0.363

*VATS* video assisted thoracoscopic surgery

There were no statistically significant differences in duration of operation and of the volume of blood loss during the operation between the two groups, and there were no statistically significant differences either in average duration of chest tube drainage (3.2(±1.8) days vs 5.0(±3.2) days, *p* = 0.104) and length of hospital stay (9.2(±5.0) days vs 11.9(±11.1) days, *p* = 0.512) after operation. There was no postoperative mortality. Postoperative complications occurred in 7 patients (30.4 %) in total. In Group A, complications occurred in 2 patients (22.2 %) in total and in both cases it was incomplete reexpansion of lung, with both cases recovering after a few months following operation. In Group B complications occurred in 5 patients (35.6 %) in total; with 1 patient with 8 days of prolonged air leak, 2 patients with postoperative bleeding with pleural blood drainage over 1000 ml within 24 h after operation (and who recovered after transfusion without reoperation), 1 patient with delayed hemothorax (which occurred on the 12^th^ day after operation and who recovered after intercostal artery embolization and pleural drainage), and finally 1 patient with bronchopleural fistula (BPF) after cavernostomy. The BPF patient was discharged from hospital with open chest tube drainage under careful monitoring and on the 48^th^ day after operation, the BPF closed spontaneously and the drainage tube was removed. After that, he was monitored for 25 months and did not develop any problem (Table 3).

**Table 3:** Surgical outcomes

	Group A	Group B	*P* value
Operation time (min)	118.4 (±48.3)	160.9 (±81.3)	0.174
Blood loss (ml)	452.2 (±640.5)	309.2 (±221.2)	0.535
Air leak (days)	0	0.86 (±2.2)	0.171
Chest tube drainage period (days)	3.2 (±1.8)	5.0 (±3.2)	0.104
Postoperative hospital stay (days)	9.2 (±4.9)	11.9 (±11.1)	0.512
Morbidity	2 (22.2 %)	5 (35.6 %)	0.657
Incomplete reexpansion	2 (22.2 %)	0 (0 %)	
Prolonged air leaks	0 (0 %)	1 (7.1 %)	
Hemorrhage	0 (0 %)	3 (21.4 %)	
BPF	0 (0 %)	1 (7.1 %)	
30-days mortality (%)	0	0	

*BPF* bronchopleural fistula

Logistic Regression analysis was used to evaluate the risk factors for complications. After univariable analysis, division of groups was not risk factors for complication incidence (*p* = 0.318) and analysis found that the possibility of the complication occurrence was higher in cases where the sex of the patient was male (OR = 8.25, *p* = 0.036) and the range of the operation was wide (wedge resection < segmentectomy < lobectomy, OR = 3.012, *p* = 0.029). In multivariate analysis none of the factors, including the division of groups, showed statistic significance (Table 4).

**Table 4:** Risk factors for postoperative morbidity by univariate logistic regression analysis

Risk factors	Odds ratio	95 % confidence interval	*P* value
Sex (male)	8.250	1.154–59.003	0.036
Age	0.997	0.947–1.050	0.906
Group (Group B)	2.625	0.395–17.458	0.318
Operation duration (min)	1.009	0.996–1.022	0.180
Intraoperative blood loss (ml)	1.000	0.998–1.002	0.910
ANC	1.000	1.000–1.000	0.632
Hemoglobin (g/dl)	1.431	0.900–2.275	0.130
Platelet count (×10^9^/L)	1.001	0.991–1.012	0.778
FEV1 (%)	0.960	0.886–1.041	0.320
Type (complex)	2.500	0.428–14.607	0.309
Extent of procedure	3.012	1.122–8.089	0.029
VATS	0.606	0.097–3.788	0.592

*ANC* absolute neutrophil count, *FEV1* forced expiratory volume in 1 s, *VATS* video assisted thoracoscopic surgery

Mean follow-up period was 692.5 days (±351.2). During follow-up periods, 2 patients in Group A died 114 days and 187 days after operation respectively, and both of them died of complications (sepsis, graft-versus-host disease) after HSCT. During the follow-up period, no recurrence of fungal disease was observed in any patients.

## Discussion

Although pulmonary mycotic infections can be caused by various types of fungi, aspergillosis and mucormycosis can be said to be the typical ones which cause localized pulmonary mycotic infections [16]. It is known that while aspergillosis can cause disease in both the immunocompetent patient and the immnucompromised patient, mucormycosis can cause disease only in the immunocompromised patient [17]. In this study, 21 of 23 patients were diagnosed with aspergillosis and 2 patients were diagnosed with mucormycosis.

When aspergillosis occurs as a localized lung disease, aspergilloma is a typical picture; it also occurs sometimes in IPA as a localized lung disease.

In cases of aspergilloma, hemoptysis is the most frequent symptom. If the patient is immunocompetent, antifungal agent is of no use and only surgical treatment is known to be effective treatment [9, 18]. Although Jewkes el al. reported that it would be better to apply surgical treatment only if hemoptypsis was detected, due to the high morbidity and mortality rate of surgery [19], many studies have reported that surgical treatment could increase the survival rate even if there were no symptoms, since in recent years the severity of the underlying lung disease has been low in many cases and surgical outcomes were getting better along with the development of operative techniques [20, 21]. Therefore, it is generally considered best to perform surgical treatment if there are symptoms, and if there are no symptoms, to perform surgical treatment only if the patient’s general condition can withstand pulmonary resection [7, 22].

In the case of IPA, since it is often not only invasive into surrounding tissue but also disseminated systemically through the whole body, initial treatment with antifungal agents is recommended. However mortality is known to be high even if treated with antifungal agents [5, 23, 24]. Even though a few studies have reported that the surgical treatment of IPA increased the survival rate, those studies are now considered inappropriate in evaluating current surgical outcomes since most of those studies evaluated patients at single centers and over a long period of time in the considerable past [11–14, 21], and surgical treatment methods have developed since then. However, in this study we evaluated patients treated in a recent comparatively short period of 3 years, and treated by recent surgical techniques at a single center, so we believe the comparison of surgical outcomes in this study would be more realistic than those of the existing studies.

While mucormycosis also has a clinical course similar to IPA, it can proceed to disseminated disease in immunocompromised patients and it has been reported that the mortality is very high when treated only with medical treatment [17]. There have been reports that surgical treatment increased survival rates in cases of localized pulmonary mucormycosis [16, 25]. However, since the disease incidence was very low and only a few studies were reported, there has been no certain established treatment. In this study, we evaluated 2 patients with the simple type of lesion and who were treated with resection (wedge resection). They were discharged from hospital without complications and without relapse for a mean 661.5 days of follow-up.

Aspergilloma can be divided into simple and complex types [15], and in the case of the complex type, the operation is known to be complicated with a high operative risk since the lung around the lesion is abnormal. In the case of IPA, even though there are some cases which look similar to the simple type on radiologic imaging, in many cases they are similar to the complex type since the disease infiltrates into the surrounding lung parenchyma, but IPA has not been divided into simple and complex types. In this study, infiltration into the surrounding lung was classified as the complex type, based on imaging study findings. In this way, both Group A and B could be divided into simple and complex types. With this division, we evaluated the distribution of types in both groups and found there was no statistical difference between the two groups. In addition, there was no difference in pulmonary function between the two groups and there was also no significant difference in the range of the surgical procedure. Therefore both groups were considered under about the same conditions and under these conditions it was considered meaningful to compare the surgical outcome.

In Group A, there were 6 cases of disease occurrence after chemotherapy, 1 case after immunotherapy, and 2 cases after HSCT. All of them were considered to have occurred after having gone through a neutropenic phase. Most of them had underlying conditions such as neutropenia, anemia, and thrombocytopenia. Delaying the operation, or giving blood transfusion were measures used to maintain the platelet count over 50(×10^9^/L) and the hemoglobin over 10.0 g/dl for the safety of operation. Nevertheless, the preoperative hemoglobin and platelet counts of Group A were significantly lower than those of Group B. Even though the average intraoperative blood loss of Group A (452.2 ml) was more than that of Group B (309.2 ml), there was no statistically significant difference. In addition in both groups there was no case of reoperation due to postoperative bleeding.

Among Group A patients, there was 1 case in which it was considered difficult to perform sugical resection due to too severe infiltration into the surrounding lung at the initial stage, but lung infiltration reduced significantly after use of antifungal agent, making it possible to perform pulmonary resection; and 6 patients out of total 9 showed a partial response so that it was possible to reduce the range of operation. In the case of IPA, studies have reported voriconazole to be most effective antifungal agent [2, 4, 6]. However, in this study, voriconazole was used only for 2 patients and amphotericin B for 4 patients, and oral antifungal agents (fluconazole, itraconazole, posaconazole) for the remaining 3 patients.

It has been reported in many studies that the surgical treatment of aspergilloma resulted in 20–30 % of morbidity and 5–10 % of mortality [7], and although there have not been many studies in the case of IPA, Nebiker et al. have reported 15–23 % morbidity and 7 % mortality [13]. In this study, morbidity was 22.2 and 35.6 % in Group A and B respectively and there was no significant difference from that of the existing studies. However in this study there was neither any case of major complication requiring management (like reoperation) nor any 30-day mortality. It could not be said that surgical treatment of hematopoietic disorder patient had more operative risk since the division of groups proved to be meaningless in the complications risk factor evaluation.

The limitations of this study were as follows. First, it was retrospective study. Second, a relatively small number of patients were included. Third, only selected patients with hematopoietic disorder underwent surgery; in other words, surgery was performed neither in cases where a higher level of surgery over pneumonectomy was required due to severe disseminated disease or wide lung infiltration, nor was it performed in cases of graft versus host disease after chemotherapy or HSCT. Surgery was not performed either if high risk factors of general anesthesia existed, namely renal failure, hepatic failure, sepsis, etc. However, surgery was performed as extensively as possible in case pulmonary resection was considered possible.

## Conclusions

In summary, the surgical treatment of localized pulmonary mycotic infection can achieve good surgical outcomes if complete resection is possible in hematopoietic disorder patients who have had immunosupression, coagulopathy, anemia and severe pulmonary infiltration.

## Abbreviations

HSCT: Hematopoietic stem cell transplantation
ABPA: Allergic bronchopulomonary aspergillosis
IPA: Invasive pulmonary aspergillosis
AML: Acute myeloid leukemia
ALL: Acute lymphoblastic leukemia
MDS: Myelodysplastic syndrome
VATS: Video-assisted thoracoscopic surgery
CT: Computed tomography
WBC: White blood cell
ANC: Absolute neutrophil count
CBC: Complete blood cell count
FEV1: Forced expiratory volume in 1 s
FVC: Forced vital capacity
RUL: Right upper lobe
BPF: Bronchopleural fistula

## References

[1] Longo DL, Fauci AS, Kasper DL, Hauser SL, Jameson JL, Loscalzo J (2012). Harrison's Principles of Internal Medicine.

[2] Walsh TJ, Anaissie EJ, Denning DW, Herbrecht R, Kontoyiannis DP, Marr KA (2008). Treatment of aspergillosis: clinical practice guidelines of the Infectious Diseases Society of America. Clin Infect Dis.

[3] Denning DW (1998). Invasive aspergillosis. Clin infect Dis.

[4] Herbrecht R, Denning DW, Patterson TF, Bennett JE, Greene RE, Oestmann JW (2002). Voriconazole versus amphotericin B for primary therapy of invasive aspergillosis. N Engl J Med.

[5] Upton A, Kirby KA, Carpenter P, Boeckh M, Marr KA (2007). Invasive aspergillosis following hematopoietic cell transplantation: outcomes and prognostic factors associated with mortality. Clin infect Dis.

[6] Denning DW, Ribaud P, Milpied N, Caillot D, Herbrecht R, Thiel E (2002). Efficacy and safety of voriconazole in the treatment of acute invasive aspergillosis. Clin Infect Dis.

[7] Passera E, Rizzi A, Robustellini M, Rossi G, Della Pona C, Massera F (2012). Pulmonary aspergilloma: clinical aspects and surgical treatment outcome. Thorac Surg Clin.

[8] Regnard JF, Icard P, Nicolosi M, Spagiarri L, Magdeleinat P, Jauffret B (2000). Aspergilloma: a series of 89 surgical cases. Ann Thorac Surg.

[9] Muniappan A, Tapias LF, Butala P, Wain JC, Wright CD, Donahue DM (2014). Surgical therapy of pulmonary aspergillomas: a 30-year North American experience. The Annals of thoracic surgery.

[10] Kim YT, Kang MC, Sung SW, Kim JH (2005). Good long-term outcomes after surgical treatment of simple and complex pulmonary aspergilloma. Ann Thorac Surg.

[11] Baron O, Guillaumé B, Moreau P, Germaud P, Despins P, De Lajartre AY (1998). Aggressive surgical management in localized pulmonary mycotic and nonmycotic infections for neutropenic patients with acute leukemia: report of eighteen cases. J Thorac Cardiovasc Surg.

[12] Salerno CT, Ouyang DW, Pederson TS, Larson DM, Shake JP, Johnson EM (1998). Surgical therapy for pulmonary aspergillosis in immunocompromised patients. Ann Thorac Surg.

[13] Nebiker CA, Lardinois D, Junker L, Gambazzi F, Matt P, Habicht JM (2012). Lung resection in hematologic patients with pulmonary invasive fungal disease. Chest.

[14] Cesaro S, Pegoraro A, Tridello G, Pillon M, Cannata E, Faggin S (2014). The role of surgery in the treatment of invasive fungal infection in paediatric haematology patients: a retrospective single-centre survey. Mycoses.

[15] Belcher JPN (1960). Surgery in broncho-pulmonary aspergillosis. Br J Dis Chest.

[16] Merritt RE, Shrager JB (2012). Indications for surgery in patients with localized pulmonary infection. Thorac Surg Clin.

[17] Bigby TD, Serota ML, Tierney LM, Matthay MA (1986). Clinical spectrum of pulmonary mucormycosis. Chest.

[18] Sagan D, Goździuk K (2010). Surgery for pulmonary aspergilloma in immunocompetent patients: no benefit from adjuvant antifungal pharmacotherapy. Ann Thorac Surg.

[19] Jewkes J, Kay PH, Paneth M, Citron KM (1983). Pulmonary aspergilloma: analysis of prognosis in relation to haemoptysis and survey of treatment. Thorax.

[20] Lee JG, Lee CY, Park IK, Kim DJ, Chang J, Kim SK (2009). Pulmonary aspergilloma: analysis of prognosis in relation to symptoms and treatment. J Thorac Cardiovasc Surg.

[21] Lejay A, Falcoz PE, Santelmo N, Helms O, Kochetkova E, Jeung M (2011). Surgery for aspergilloma: time trend towards improved results?. Interact Cardiovasc Thorac Surg.

[22] Sagan D, Goździuk K, Korobowicz E (2010). Predictive and prognostic value of preoperative symptoms in the surgical treatment of pulmonary aspergilloma. J Surg Res.

[23] Nucci M, Nouér SA, Cappone D, Anaissie E (2013). Early diagnosis of invasive pulmonary aspergillosis in hematologic patients: an opportunity to improve the outcome. Haematologica.

[24] Salman N, Törün SH, Budan B, Somer A (2011). Invasive aspergillosis in hematopoietic stem cell and solid organ transplantation. Expert Rev Anti-Infect Ther.

[25] Tedder M, Spratt JA, Anstadt MP, Hegde SS, Tedder SD, Lowe JE (1994). Pulmonary mucormycosis: results of medical and surgical therapy. Ann Thorac Surg.

